# Epidermal stem cells undergo age-associated changes

**DOI:** 10.18632/aging.100530

**Published:** 2013-01-15

**Authors:** Jason Doles, William M. Keyes

**Affiliations:** Centre for Genomic Regulation (CRG), Universitat Pompeu Fabra (UPF), Barcelona, 08003, Spain

The causes underlying aging remain poorly understood. One prominent theory is that a decrease in stem cell function over time plays a significant role in tissue aging, which ultimately manifests at the organismal level. This could be through cell-intrinsic alterations in the stem cell pool, cell-extrinsic changes affecting stem cell function, or a combination of both. However, the noticeable exception to this idea was the fact that the skin, which contains some of the most amenable and best-studied stem cell populations and which progressively loses its ability to maintain tissue homeostasis with age, had no previously documented age-associated changes in stem cell function [1].

Recent work has now uncovered a subset of epidermal stem cells in the hair follicle bulge that undergoes significant changes during normal aging [2]. Using the well-characterized Keratin-15-GFP reporter mouse [3] to study and isolate stem cells during aging, this study identified increases in stem cell number but decreases in functional capacity of this population over time, and advances the hypothesis that broader age-associated stem cell alterations contribute significantly to skin aging.

In light of this finding, it is important to keep in mind the diverse heterogeneity of stem cell populations present in the mammalian epidermis. This heterogeneity not only applies to functionality, but also manifests in a compartmentalized fashion, with discrete sets of stem cells occupying distinct anatomical niches, including the interfollicular epidermis (IFE), the hair follicle (HF) bulge, and the isthmus region at the interface of the IFE and HF. It is this heterogeneity, in both location and function that likely masked previous attempts to identify aging stem cell changes in skin [1, 4]. Indeed, this present study identified changes in the sub-population of HF stem cells that label positive for the Keratin-15-GFP reporter, while co-staining of this population with the classical stem cell markers CD34 and a6-integrin further identified these as aging stem cells. It will be interesting to interrogate these findings further to see how changes in the Keratin-15-GFP population identified in this study might affect the functional behavior, fate and cellular hierarchy in other skin stem cell populations and their derivatives.

This study identified that the Keratin-15-GFP cells accumulate in number and broadly upregulate a gene-expression signature of undifferentiated stem cells, suggesting that there may be a functional block in the differentiation capacity of these cells in vivo. However, an alternative hypothesis may be that there is an active de-differentiation process in more committed cells in the aged tissue, thus compensating for decreased functional capacity [5]. An examination of differentiation potential at the transcript level suggests that aged Keratin-15-GFP cells may be restricted in their ability to differentiate along non-core hair follicle lineages, supporting both of these ideas. In addition, functional assays in this study demonstrated an inherent inability of these stem cells to tolerate stress, such as gamma-irradiation and strong proliferative stimuli such as treatment with the phorbol ester TPA or Jak-Stat inhibitors. Together, this suggests that there are likely significant cell-intrinsic changes contributing to the impaired functional capacity during aging.

Another striking finding of this study was the pronounced increase in epidermally-derived cytokine/ chemokine expression. Functional studies demonstrated that certain factors which increase their expression during aging could functionally impair stem cell function, adding to the body of work that age-associated inflammatory signals might contribute to aging by altering stem cell function [6]. However, it is particularly interesting that the signature of the aged epidermis bears strong resemblance to the senescence-associated secretory phenotype (SASP) which has been suggested to play a major role in tissue aging but which has yet to be shown to impact stem cell function directly [7]. This study strongly supports the concept that the accumulation of senescent cells described in normal aging skin [7] may be a potent factor in tissue aging by inhibiting stem cell function and disrupting homeostasis.

In addition, it is interesting to note that both the aged epidermis and stem cell compartment exhibit a significant increase in phospho-Stat3 levels, which may have distinct functional consequences in each setting. Indeed, studies have shown that skin-specific ablation of Stat3 has deleterious consequences for skin and hair follicle maintenance inducing aging-like phenotypes [8]. Furthermore, phospho-Stat3 can be pro-tumorigenic, suggesting that the aged stem cells, the suggested cell of origin in squamous cell carcinoma, might already be more prone to tumor initiation and that unknown tumor suppressive mechanisms could be acting in opposition to this pressure and contribute to aging. However, phospho-Stat3 is also a critical pluripotency factor, facilitating reprogramming and stemness [9] which, when expressed in a committed transit amplifying cell may be able to drive de-differentiation and stem cell accumulation. It will be interesting to tease apart the cell-type specific roles for Stat3 in the aging epidermis.

The finding that there are significant quantitative and functional alterations in stem cell populations has been demonstrated for many tissues, including the hematopoietic, nervous, gastrointestinal and muscle systems. Adding the skin to this list should remove any doubt about the functional significance of age-related changes in stem cell populations and the link with organismal aging.
